# [Retracted] Bindarit reduces the incidence of acute aortic dissection complicated lung injury via modulating NF-κB pathway

**DOI:** 10.3892/etm.2026.13349

**Published:** 2026-11-04

**Authors:** Zhiyong Wu, Jinxing Chang, Wei Ren, Zhipeng Hu, Bowen Li, Huagang Liu

Exp Ther Med 14: 2613-2618, 2017; DOI: 10.3892/etm.2017.4830

Following the publication of the above paper, a concerned reader has drawn the Editor's attention to the fact that this paper contained data that were shared with another article featuring several of the named authors in common that was submitted during the same week of the same year to the journal *American Journal of Translational Research.* Specifically, various of the data panels showing the results of histological/immunohistochemical experiments in Figs. 2 and 3 on p. 2616 were also featured in the other paper, sometimes apparently to show the same experimental results, although not universally so. In addition, an overlapping panel of data was noted comparing Fig. 2B with the “HE staining / BAPN+AngII” data panel in Fig. 3, suggesting that these data were derived from the same original source. A further aspect of potential concern regarded the identification of tabulated data in Tables I and II of the above paper that were identical to data reported in the *American Journal of Translational Research* paper.

After having conducted an independent investigation of this paper in the Editorial Office, the Editor of *Experimental and Therapeutic Medicine* has determined that it should be retracted from the Journal on account of a lack of confidence in the authenticity of the presented data. The authors were asked for an explanation to account for these concerns, but the Editorial Office did not receive a reply. The Editor regrets any inconvenience that has been caused to the readership of the Journal.

